# Profile of micronucleus frequencies and DNA damage in different species of fish in a eutrophic tropical lake

**DOI:** 10.1590/S1415-47572009005000009

**Published:** 2009-01-17

**Authors:** Cesar K. Grisolia, Carla L. G. Rivero, Fernando L. R. M. Starling, Izabel C. R. da Silva, Antonio C. Barbosa, Jose G. Dorea

**Affiliations:** 1 Departamento de Genética e Morfologia, Instituto de Ciências Biológicas, Universidade de Brasília, Brasília, DFBrazil; 2 Companhia de Água e Esgoto de Brasília, Brasília, DFBrazil; 3 Laboratório de Biologia Molecular, Departamento de Biologia Celular, Universidade de Brasília, Brasília, DFBrazil; 4 Instituto de Química, Universidade de Brasília, Brasília, DFBrazil; 5 Departamento de Nutrição, Universidade de Brasília, Brasília, DFBrazil

**Keywords:** genotoxicity, fish biomonitoring, eutrophic lake, micronucleus, comet assay, cytotoxicity

## Abstract

Lake Paranoá is a tropical reservoir for the City of Brasilia, which became eutrophic due to inadequate sewage treatment associated with intensive population growth. At present, two wastewater treatment plants are capable of processing up to 95% of the domestic sewage, thereby successfully reducing eutrophization. We evaluated both genotoxic and cytotoxic parameters in several fish species (*Geophagus brasiliensis*, *Cichla temensis*, *Hoplias malabaricus*, *Astyanax bimaculatus lacustres*, *Oreochromis niloticus*, *Cyprinus carpio* and *Steindachnerina insculpita*) by using the micronucleus (MN) test, the comet assay and nuclear abnormality assessment in peripheral erythrocytes. The highest frequencies of MN were found in *Cichla temensis* and *Hoplias malabaricus*, which were statistically significant when compared to the other species. However, *Steindachnerina insculpita* (a detritivorous and lake-floor feeder species) showed the highest index of DNA damage in the comet assay, followed by *C. temensis* (piscivorous). Nuclear abnormalities, such as binucleated, blebbed, lobed and notched cells, were used as evidence of cytotoxicity. *Oreochromis niloticus* followed by *Hoplias malaricus*, ominivorous/detritivotous and piscivorous species, respectively, presented the highest frequency of nuclear abnormalities, especially notched cells, while the herbivorous *Astyanax bimaculatus lacustres* showed the lowest frequency compared to the other species studied. Thus, for biomonitoring aquatic genotoxins under field conditions, the food web should also be considered.

## Introduction

Lake Paranoá is a tropical reservoir, built in 1959 along with the city of Brasília, the new capital of Brazil. Within a decade, the lake became eutrophic due to inadequate sewage treatment associated with high population growth (Altafin *et al.*, 1995). With the construction of two wastewater treatment plants, capable of processing up to 95% of domestic sewage to the tertiary level, the eutrophization process was greatly reduced (Cavalcanti *et al.*, 1997). Currently, a diversity of fish flourish in Lake Paranoá, including native and exotic species, creating an opportunity for comparative genotoxicity studies. Indeed, *Cyprinus carpio*, *Oreochromis niloticus* and *Tilapia rendalli* specimens from Lake Paranoá have previously been studied for their sensitivity to different clastogens, such as bleomycin, cyclophosphamide, mitomycin C and 5-fluorouracyl. These three fish species were sensitive to all clastogens, as demonstrated by micronucleus induction (Grisolia and Cordeiro, 2000).

Different species of aquatic organisms, such as clams, mussels, fish, and amphibians, are used to investigate the genotoxicity of waters (Burgeot *et al.*, 1995; Ralph and Petras, 1997; Buschini *et al.*, 2004; Lemos *et al.*, 2005; Souza and Fontanelli, 2006), and the micronucleus test (MN) is one of the simplest and quickest tests for biomonitoring the genotoxicity of aquatic environments. The MN method using peripheral blood smears of fish is widely used and recommended (Hooftman and Raat, 1982; Manna and Sadhukhan, 1986; Jha *et al.*, 1994; Çavas and Ergene-Gozukara, 2003). Some studies using the MN test in fish have found it to be an excellent tool for assessing genotoxicty of waterborne substances (Mersch and Beauvais, 1997; Matsumoto and Cólus, 2000; Grisolia and Starling, 2001; Gustavino *et al.*, 2001; Russo *et al.*, 2004; Porto *et al.*, 2005). Alkaline single-cell gel electrophoresis (SCGE), or comet assay, is a rapid, simple and sensitive procedure for quantifying DNA lesions in individual cells. It is used for environmental monitoring and for detecting DNA damage in aquatic animals such as fish, clams, shellfish and mussels (Hayashi *et al.*, 1998; Mitchelmore and Chipman, 1998; Russo *et al.*, 2004; Andrade *et al.*, 2004; Belpaeme *et al.*, 2004). The aim of the present study was to investigate the profile of genotoxicity and cytotoxicity biomarkers in different species of fish from Lake Paranoá, a man-made eutrophic tropical reservoir, and to evaluate the difference in sensitivity among species, as well as between the MN and the comet assays.

**Figure 1:**
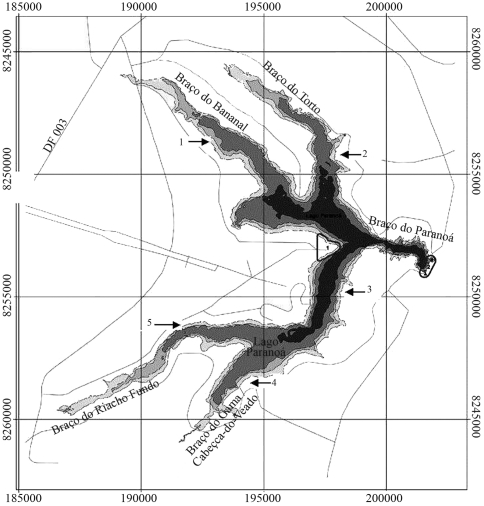
Map of Lake Paranoá showing the five sample sites (arrows).

## Methods

### Characteristics of Lake Paranoá

Lake Paranoá covers 38 km^2^ and is located between 15°43'60'' and 15°50'50" S, and 47°47'30'' and 47°55'50" W. Five sampling sites were selected, as shown on the map (Figure 1). The watershed of the lake is characterized by a low level of industrial and agricultural activity. Previous studies on heavy metals, pesticides and pathogenic bacteria have demonstrated the low levels of such contaminants in the water, sediment and fish from Lake Paranoá (CAESB, 1996). The nutrient input from domestic sewage is the main source of pollution in Brasília, a city that has no chemical industry. Lake Paranoá is typically eutrophic, with phosphorus representing the limiting nutrient for algal growth (Altafin *et al.*, 1995). For each sample site, measurements of biochemical oxygen demand (BOD), chemical oxygen demand (COD), total phosphorous (TP) and total nitrogen (TN) were made to show the eutrophization level of the lake. These data were obtained by a routine monitoring program carried out by the Brasília Municipal Drinking Water and Sewage Corporation (CAESB) (Table 1). A total of 205 specimens were captured by appropriate cast-nets at the five sites (Figure 1). Four to six drops (0.1 mL approximately) of peripheral blood were drawn from the gills using heparinized syringes, and immediately smeared onto two slides, for MN and nuclear abnormalities analysis, respectively; another two drops were placed in a microtubule with 500 μL of HAM-F10 serum (Difco, Ind., Detroit,) for the comet assay. This procedure was chosen because it is suitable for these species and does not require sacrificing the animals tested. After sampling, the specimens were released back into the lake. The studied species *Geophagus brasiliensis* (acará, omnivorous/floor-feeder), *Cichla temensis* (tucunaré, piscivorous), *Hoplias malabaricus* (traíra, piscivorous), *Astyanax bimaculatus lacustres* (lambari, predominantly herbivorous and omnivorous) and *Steindachnerina insculpita* (saguiru, detritivorous) are native species. *Oreochromis niloticus* (tilápia, omnivorous and detritivorous) and *Cyprinus carpio* (carpa, predominantly algivorous and omnivorous) are exotic species from Africa and Asia, respectively.

### Micronucleus test

The slides were fixed in methanol for 15 min, and the smears were stained with Giemsa (5%). A total of 3000 erythrocytes were examined for each fish, under an immersion objective (1000x). The micronucleus test was carried out as described by Hooftman and Raat (1982) for fish erythrocytes. The criteria for the identification of fish micronucleated erythrocytes were as follow: (a) the MN had to be smaller than one-third of the main nuclei; (b) the MN could not touch the main nuclei; (c) the MN could not be refractive and should have the same color and staining intensity as the main nuclei. The presence of nuclear abnormalities in peripheral erythrocytes, as proposed by Carrasco *et al.* (1990), was used as a biomarker of cytogenotoxicity. According to their shape, the nuclei were classified as blebbed, lobed, notched and binucleated. One thousand cells were scored per fish to calculate the percentage of cells with heteromorphic nuclei. The different frequencies of classes of nuclear deformities observed in treatments and control were statistically analyzed by Mann Whitney's non-parametric test – *U*, with a significance level of 95%. Micronuclei and nuclear abnormalities were counted in peripheral erythrocytes of the sample slide.

### Alkaline single cell gel electrophoresis (comet assay)

This assay was performed as described by Singh *et al.* (1988), with some modifications. The cell suspension sampled in the microtubule was mixed with 120 μL low-melting agarose (37 °C). Then, 500 μL of the erythrocyte-agarose suspension were placed onto a fully frosted slide pre-coated with standard agarose (1.5%) and covered with a coverslip. The slides were then placed on ice for 15 min to allow complete agarose polymerization and afterwards in a chilled lysing solution (NaCl 2.5 M; EDTA 100 mM; Tris 10 mM; N-laurolyl-sarcosine 1%; Triton-X 1%; DMSOn 10%; pH = 10). Then the slides were placed on a horizontal gel electrophoresis platform and covered with a chilled alkaline solution consisting of 300 mM NaOH and 1 mM Na_2_EDTA (pH = 13), left in the dark at 4 °C for 30 min and then the DNA was electrophoresed at 4 °C in the dark for 30 min at 25 V and approximately 350 mA. Then the slides were gently rinsed twice with 400 mM Tris (pH = 7.5) to neutralize the alkali. Each slide was stained with 30 μL of 20 μg/mL ethidium bromide and covered with a coverslip. One hundred cells from each replicate were randomly chosen (50 from each duplicate slide), and analyzed under an optical fluorescence microscope (Axioskop-2, Carl Zeiss), with a 510-560 nm filter and a 590 nm barrier filter, with a magnification of 400x. For damage index calculation, cells were sorted into four classes, according to tail size. The damage index (DI) is the sum of classes of the 100 cells analyzed per fish, and may vary from 0 (all cells undamaged – 0X100) to 400 (all cells highly damaged – 4X100). The damage index is based on the length of migration and on the amount of DNA in the tail, and it is considered a sensitive measurement of detectable DNA damage. Statistical analysis was carried out with the MINITAB program, using the ANOVA parametric test and Tukey's parametric linear correlation, with a significance level of 95%. To quantify the damage to the DNA, the following formula was used:


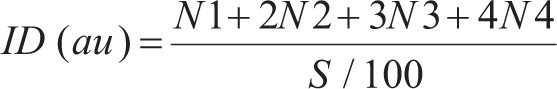


where *ID* = index damage DNA, *au* = arbitrary unit, *N*1 - *N*4 = nucleoids in levels 1, 2, 3 and 4, *S* = number of nucleoids analyzed, including level 0.

## Results

Table 2 shows the fish species captured, numbers of fish sampled at each site, and total number of species studied. *Cichla temensis* (tucunaré) and *Hoplias malabaricus* (traíra), both piscivorous species, presented the highest means of MN, 1.86 and 1.80, respectively (Table 3). Both species, when compared with all others, presented statistically significant differences in the MN frequencies (Mann-Whitney, p < 0.05). In *Geophagus brasiliensis* (acará), *Oreochromis niloticus* (tilápia), *Cyprinus carpio* (carpa) and *Steindachnerina insculpita* (saguiru), the MN frequencies were low, with no statistical differences among them (Mann-Whitney, p > 0.05). In the cytotoxicity evaluation based on nuclear abnormalities in peripheral erythrocytes, *O. niloticus* presented the highest frequency compared to all other species (Mann-Whitney, p < 0.05), whereas *Astyanax bimaculatus lacustres* presented the lowest frequency of nuclear abnormalities (Mann-Whitney, p < 0.05, Table 3). *S*. *insculpita,* a native floor-feeder species, presented the highest DNA damage index (comet assay), statistically different from all other species studied, followed by *C. temensis* (piscivorous). *O. niloticus* and *C. carpio* presented the lowest DNA damage indexes (Table 4). There was no relationship between the total number of fish sampled and the number of fish analyzed by comet assays, because some comet slides were lost due to methodological problems. No differences were observed in MN frequency and DNA damage index among the five sample sites shown on the map of Lake Paranoá (Figure 1). These fish species showed a wide range of DNA damage indexes, with a distribution going from 37.32 ± 43.63 up to 109.11 ± 64.33, considering *O. niloticus* and *S. insculpita*, respectively (Tukey's test and ANOVA, p < 0.05).

## Discussion

Given the fact that all species were exposed to the same source of cytotoxins and that their feeding and other habits are known, the piscivorous specimens showed a higher background level of micronuclei. In the comet assay, *S. insculpita* (omnivorous/floor-feeder) followed by *C. temensis* (piscivorous) presented the highest DNA damage indexes, while *O. niloticus* (omnivorous/detritivorous) followed by *C. carpio* (algivorous) presented the lowest ones. Furthermore, *O. niloticus* (omnivorous) presented the highest frequency of nuclear abnormalities, followed by *H. malabaricus* and *C. temensis (both* piscivorous). *A. bimaculatus lacustres*, an herbivorous species, showed the lowest frequency of nuclear abnormalities. In our previous study (Grisolia and Starling, 2001), the MN frequencies observed in *C. carpio*, *O. niloticus* and *T. rendalli* from Lake Paranoá were very low, even considering that these fish species were sampled near the disposal site of a wastewater treatment plant, shown in Figure 1 (arrows 1 and 5). Fish are suitable organisms for such tests because they are bioconcentrators, and many contaminants, even at low concentrations, endanger their physiology and survival (Goksoyr *et al.*, 1991; Miracle and Ankley, 2005). The MN test is often applied in combination with single cell gel electrophoresis for genetic monitoring of toxic chemicals in aquatic environments. However, data using the comet assay in fish are still being accumulated, since this technique is more recent than the MN test. In recent years, the detection of cytogenotoxicity damages in fish peripheral erythrocytes has also been introduced as a method for investigating changes in interphase cells. In these studies, lobed, blebbed and notched nuclei and binuclei were considered biomarkers of cytogenotoxicity Ayllon and Garcia-Vazquez, 2000; Palhares and Grisolia, 2002; Çavas and Ergene-Gozukara, 2003). Lake Paranoá is considered wholly eutrophized, with two hypereutrophic sites (Figure 1, arrows 1 and 5) around discharges of wastewater treatment plants. Top-chain and detritivorous fishes bioaccumulate and concentrate waterborne pollutants, reaching levels that can show genotoxicity. This study shows that there are variations in the genotoxicity profile among different fish species living in the same environment. No relationship was observed among the MN frequencies, nuclear abnormalities and comet assay results, once increased MN frequencies were observed in traíra and tucunaré, comet induction only in saguiru, and nuclear abnormalities only in tilápia fishes. Barbosa *et al.* (2003) demonstrated, in freshwater fishes from the Amazon basin, the bioaccumulation of mercury related to the trophic level. They found higher concentrations of mercury in the tertiary consumer, such as *Cichla* spp and *Serrasalmus* spp*,* followed by omnivorous fish. The same process was observed with regard to many other pollutants, such as organochlorine pesticides and polychlorobiophenyls (PCBs), which accumulate along food chains and reach the highest concentrations in the tissues of top predators (Livingstone, 1993; Kelly *et al.*, 2007). Caldas *et al.* (1999) studied organochlorine pesticide residues in water, sediment and fish from Lake Paranoá. They found significantly higher residue levels of DDT in *S. insculpita* (omnivorous/floor-feeder) and *C. temensis* (piscivorous) than in other herbivovous species. Some species, such as *A. bimaculatus lacustres,G. brasiliensis* and *C. carpio*, can change their food habits depending on the environment. *O. niloticus*, known as omnivorous, also behaves as detritivorous. Thus, besides food habits, other changes in behavior due to a stressful environment should be considered, in order to define fish species as a better bioindicator of aquatic pollution. The differences observed in the baseline frequencies of DNA damage and micronuclei among the species living in the same environment prove that we should be aware of the differential sensitivity of aquatic organisms to genotoxic agents and their responses to them, and of their relationships in the aquatic ecosystem.

## Figures and Tables

**Table 1:** Limnological features of Lake Paranoá. Results of water quality analysis from the sampled sites, using physicochemical parameters (CAESB).

	BOD mg/L	COD mg/L	TP μg/L	TN μg/L	Chlorophyll μg/L
Site 1	12.2	33.0	1.2	9.7	92.2
Site 2	8.5	31.0	0.4	5.1	80.2
Site 3	7.6	34.0	0.3	5.5	68.5
Site 4	6.8	32.0	0.3	9.8	70.6
Site 5	11.0	36.0	0.8	11.2	112.4

BOD = biochemical oxygen demand. COD = chemical oxygen demand. TP = total phosphorus. TN = total nitrogen.

**Table 2:** Number of fish species sampled at each site of Lake Paranoá.

Sites/species (common name)	*G. brasiliensis* (cará)	*C. temensis* (tucunaré)	*H. malabaricus* (traíra)	*A. b. lacustres* (lambari)	*O. niloticus* (tilápia)	*C. carpio* (carpa)	*S. insculpita* (saguiru)
Site 1	10	15	10	14	16	3	8
Site 2	3	3	5	5	5	3	5
Site 3	8	4	4	5	5	3	8
Site 4	5	3	4	7	3	8	4
Site 5	4	3	3	3	4	5	10

**Table 3:** Means and standard deviations of micronuclei and means of different classes of nuclear abnormalities counted in the species from Lake Paranoá studied.

	*Geophagus brasiliensis* (acará)	*Cichla temensis* (tucunaré)	*A. b. lacustres* (lambari)	*Hoplias malabaricus* (traíra)	*Oreochromis niloticus* (tilápia)	*Cyprinus carpio* (carpa)	*Steindachnerina insculpita* (saguiru)
Micronucleus	0.86 ± 1.24	1.86 ± 1.75^a^	1.13 ± 2.14	1.80 ± 1.30^a^	0.87 ± 1.23	0.94 ± 1.59	0.71 ± 0.92
Binucleated cells	0.16 ± 0.63	0.34 ± 0.83	0.65 ± 1.26	0.20 ± 0.65	0.79 ± 1.38	0.35 ± 0.78	0.12 ± 0.33
Blebbed cells	2.79 ± 2.51	5.21 ± 3.71	5.30 ± 3.54	0.91 ± 1.24	10.50 ± 8.09^b^	1.41 ± 1.77	1.29 ± 1.96
Lobed cells	1.58 ± 1.90	1.00 ± 1.12	1.35 ± 1.78	0.41 ± 0.58	10.00 ± 8.03^b^	1.70 ± 3.31	0.41 ± 0.92
Notched cells	1.33 ± 2.03	2.60 ± 2.29	4.90 ± 5.19	0.37 ± 0.64	13.00 ± 7.61^b^	2.05 ± 1.78	1.45 ± 1.47

^a^p < 0.05, MN frequencies, Mann Whitney – *U*; ^b^p < 0.05, nuclear abnormalities, Mann Whitney – *U*.

**Table 4:** Index of DNA damage assessed by comet assay for all species studied from the five monitoring sites (Mean ± SD).

Species	Total of fish analyzed	DNA damage index
*Geophagus brasiliensis*	15	55.53 ± 62.01
*Cichla temensis*	16	89.44 ± 39.52
*A. bimaculatus lacustres*	25	40.76 ± 61.24
*Oreochromis niloticus*	22	37.32 ± 43.63
*Steindachnerina insculpita*	18	109.11 ± 64.33*
*Hoplias malabaricus*	10	80.35 ± 29.80
*Cyprinus carpio*	14	38.25 ± 23.44

* significant; Tukey's parametric linear correlation, significance 95%.
